# Knowledge Attitude and Practice (KAP) Study of Yellow Fever among International Students of Tehran University of Medical Sciences, Iran

**Published:** 2019-07

**Authors:** Fatima Mahmud MUHAMMAD, Hamidreza BASSERI, Reza MAJDZADEH, Khandan SHAHANDEH, Abbas Rahimi FOROUSHANI

**Affiliations:** 1. Department of Epidemiology and Biostatistics, School of Public Health, Tehran University of Medical Sciences, Tehran, Iran; 2. Department of Medical Entomology and Vector Control, School of Public Health, Tehran University of Medical Sciences, Tehran, Iran; 3. Deputy of Research and Technology, Tehran University of Medical Sciences, Tehran, Iran

## Dear Editor-in-Chief

Yellow fever (YF) is among the vector-borne diseases listed in the International Health Regulation by WHO. The IHR is an international legal instrument that is binding on 196 countries across the globe, including all members of WHO. According to a report of IHR, the number of YF cases has increased over the past years due to the declining population immunity to infection, deforestation, urbanization, population movements, and climate change. The virus is endemic in tropical areas of the world; these include Africa, Central, and South America. There are an estimated 200,000 cases of YF, causing 30,000 deaths worldwide each year with 90% occurring in Africa. About 47 countries in the world are affected, in which 34 of them in Africa, Central and South America while 13 are either endemic or have regions that are endemic for the disease (1–3). YF is one of the diseases that many countries asked for proof of vaccination from international travelers since it can be spread from endemic countries to others via passengers or immigrants. Until now, there is no known cure for it. However, potent vaccines exist to provide protection for up to ten years (1, 2, 4). The most common signs and symptoms are fever, muscle pain with prominent backache, headache, loss of appetite, and nausea or vomiting. In most cases, symptoms disappear after 3 to 4 d. Good and early supportive treatment in hospitals improves survival rates (1–3, 5).

The study objective was to determine the knowledge, attitude, and practice of YF among international students of Tehran University of Medical Sciences, Tehran, Iran in 2015. The design was cross-sectional and questionnaire-based. The questionnaires were in two forms: Self-administered questionnaire for those students in the school at the time of data collection and electronic questionnaire through their e-mail for those students who were not available or out of the country at that time. The data collected were analyzed using SPSS version 22 (Chicago, IL, USA). Participants and their contacts were identified through the registry office of Global Strategies for International Affairs (GSIA).

A non-probability sampling technique and convenience sampling were used. Validity and reliability of the questionnaire were examined. Reliability test was assessed by Internal consistency, Cronbach’s alpha (The result for Cronbach’s alpha was 0.689 for knowledge, Attitude was 0.746 and practice was 0.713), the intracluster correlation was checked to measure the internal consistency of the questions (ICC for knowledge was 0.587, for Attitude was 0.448 and for practice was 0.571).

The ethical approval was obtained from the Tehran University of Medical Sciences, Tehran, Iran. Overall, 140 Questionnaires were distributed among the students out of which 124 were filled representing response rate of 88.5%.

Table 1 demonstrates the knowledge of participants about YF. Generally, Male participants had more knowledge about YF compare to female participants, but we cannot conclude that males are more knowledgeable about YF but male comes from countries where YF is endemic. Students studying public health courses have more knowledge about YF compared to students studying basic and clinical sciences. They study courses that are related to the community health, which shows preventive practices. In addition, we noticed some differences in mean between Ph.D. and MD with also bachelors. Table 2 shows the attitude of participants about YF and prevention practice.

**Table 1:** The results for knowledge by some demographic variables

***Variable***	***Characteristics***	***Numbers (n)***	***Mean (%)***	***SD (%)***
Gender	Male	78	62.222	20.74143
	Female	46	51.7391	20.08308
*P*-value = 0.007				
	Basic sciences	17	65.82	3.70612
Field of study	Clinical Sciences	86	54.1085	2.93626
	Public health	21	69.5238	2.80306
*P*-value = 0.0003				
Level of study	PhD	29	70.5747	17.4138
	MSc/MPH	29	62.9885	22.15287
	DDS/MD	50	52.6667	18.23256
	Bachelors	16	45.4167	45.4167
*P*-value = 0.000				
Region	Africa	37	72.0721	15.05856
	East Mediterranean	72	51.3389	21.30155
	Others	15	57.7778	2.734888
*P*-value = 0.000				
	20yrs below	53	48.8050	17.62737
Age group	21–30yrs	39	64.6154	21.95976
	31yrs above	32	66.4583	19.26866
*P*-value = 0.000				

**Table 2:** Attitude and practice for some demographic variable

***ATTITUDE***			
***Variable***	***Characteristics***	***Numbers (n)***	***Mean (%)***
Gender	Male	46	51.33
	Female	77	69.09
*P*-value = 0.008			
Field of study	Basic sciences	17	68.06
	Clinical sciences	86	56.29
	Public health	21	80.19
*P*-value = 0.014			
	Africa	37	77.86
Region	East Mediterranean	71	54.04
	Others	15	60.57
*P*-value = 0.003			
PRACTICE			
Variable	Characteristics	Numbers (n)	Mean (%)
Region	Africa	37	79.59
	East Mediterranean	52	53.56
	Others	15	63.27
*P*-value = 0.001			

The students of Public Health School showed also high positive attitude compare to students from another field. In terms of region students from African region, have positive attitudes towards YF compared to students from Asia. Regarding practice, male students have better preventive practices than females. Majority of the students were not vaccinated against YF. However, only 20% of students from African countries were vaccinated.

## References

[1] WHO (2016). World Health Organization Yellow fever fact sheet. assessed 9^th^ October 2017.

[2] Valdes MAS (2017). Yellow Fever: It worth a review in the current epidemiological context. MediSur, 15(1):63–70.

[3] WHO (2015). Vaccines and vaccination against yellow fever: WHO Position Paper, June 2013-Recommendations. Vaccine, 33(1):76–7. 24852721 10.1016/j.vaccine.2014.05.040

[4] Monath TP (2001). Yellow fever: an update. Lancet Infect Dis, 1(1):11–20. 11871403 10.1016/S1473-3099(01)00016-0

[5] Garske T Van Kerkhove MD Yactayo S (2014). Yellow Fever in Africa: Estimating the Burden of Disease and Impact of Mass Vaccination from Outbreak and Serological Data. PLoS Med, 11(5): e1001638. 24800812 10.1371/journal.pmed.1001638 PMC4011853

